# The Assessment of Daily Energy Expenditure of Commercial Saturation Divers Using Doubly Labelled Water

**DOI:** 10.3389/fphys.2021.687605

**Published:** 2021-05-26

**Authors:** Sanjoy K. Deb, Eimear Dolan, Catherine Hambly, John R. Speakman, Olav Eftedal, Mohammed Gulrez Zariwala, Ingrid Eftedal

**Affiliations:** ^1^Centre for Nutraceuticals, School of Life Sciences, University of Westminster, London, United Kingdom; ^2^Department of Circulation and Medical Imaging, Faculty of Medicine and Health Sciences, Norwegian University of Science and Technology, Trondheim, Norway; ^3^Applied Physiology and Nutrition Research Group, University of São Paulo, São Paulo, Brazil; ^4^Institute of Biological and Environmental Sciences, University of Aberdeen, Aberdeen, United Kingdom; ^5^Centre of Excellence in Animal Evolution and Genetics, Chinese Academy of Sciences, Kunming, China; ^6^Equinor ASA, Stavanger, Norway; ^7^Faculty of Nursing and Health Sciences, Nord University, Bodø, Norway

**Keywords:** saturation diving, extreme environment, hyperbaric, energy expenditure, doubly labelled water

## Abstract

Commercial saturation divers are exposed to unique environmental conditions and are required to conduct work activity underwater. Consequently, divers’ physiological status is shown to be perturbed and therefore, appropriate strategies and guidance are required to manage the stress and adaptive response. This study aimed to evaluate the daily energy expenditure (DEE) of commercial saturation divers during a 21-day diving operation in the North Sea. Ten saturation divers were recruited during a diving operation with a living depth of 72 metres seawater (msw) and a maximum working dive depth of 81 msw. Doubly labelled water (DLW) was used to calculate DEE during a 10-day measurement period. Energy intake was also recorded during this period by maintaining a dietary log. The mean DEE calculated was 3030.9 ± 513.0 kcal/day, which was significantly greater than the mean energy intake (1875.3 ± 487.4 kcal; *p* = 0.005). There was also a strong positive correction correlation between DEE and total time spent performing underwater work (*r* = 0.7, *p* = 0.026). The results suggested saturation divers were in a negative energy balance during the measurement period with an intraindividual variability in the energy cost present that may be influenced by time spent underwater.

## Introduction

Saturation diving is an extreme environmental occupation, with extended exposure to a confined, hyperbaric, hyperoxic environment to allow subsea activity for prolonged periods. The hyperbaric environment places unique demands on the human body that challenges the physiological, cognitive, and physical functions of professional saturation divers. These challenges may cause immunosuppression, reduce body mass, and alter the gut microbiome environment (Brubakk et al., 2014; Yuan et al., 2019). Although longitudinal studies to assess the chronic health-related implications of saturation diving is sparse, purported long-term consequences include reduced musculoskeletal health and impaired cognition (Ross et al., 2007; Brubakk et al., 2014).

Given the potential health-related consequences of prolonged exposure to this environmental stressor, there is a clear need to develop strategies to support acclimatisation, adaptation, and management of physiological perturbations that these unique environmental conditions bring. Appropriate and targeted nutritional interventions represent some such potential mitigation approach and are believed to play a vital role in managing a saturation divers physiology, health and wellness (Deb et al., 2016). For example, oral antioxidant supplementation has been reported to reduce hepatic oxidative damage during saturation diving (Ikeda et al., 2004). Furthermore, commercial saturation diving results in changes to the gut microbiome by reducing Bifidobacterium and short-chain fatty acid (Yuan et al., 2019), both of which can also be modulated by nutritional intervention (Ojeda et al., 2016). Despite emerging research highlighting the importance of nutrition for saturation divers’ health, there remains a gap in knowledge on this occupation’s unique dietary needs (Deb et al., 2016).

Energy requirements are a fundamental component of dietary guidelines, as it provides a quantitative assessment of physiological and behavioural energy cost of an individual within their environment (Speakman, 1999). Commercial saturation divers self-reported their daily activity within the chamber’s confinement to be low (Dolan et al., 2016), with the main activity (and therefore activity-related energy expenditure) occurring during underwater excursions. This underwater activity’s energy requirements are unknown; although, reports suggest that this can vary between diving operations depending on the tasks performed (Gernhardt and Lambertsen, 2002; Dolan et al., 2016). Taken together, the changes in daily physical activity in the chamber, the atmospheric conditions and the energy cost of underwater activity may all theoretically influence daily energy expenditure (DEE) during commercial saturation diving. However, to date, an accurate assessment of DEE during the real-life offshore work environment has not been undertaken. Establishing the energy requirements of saturation diving is essential to formulate appropriate nutritional guidelines to support occupational divers’ health and well-being (Deb et al., 2016). Therefore, the purpose of this study was to determine the average DEE of occupational saturation divers who are undertaking a 21-day commercial dive in the North Sea using the gold standard energy assessment technique of doubly labelled water (DLW).

## Methods

### Participants and Saturation Dive

Ten operational and medically certified divers were recruited with the following characteristics (mean ± SD), age: 47 ± 8.4 years; height: 180.4 ± 7.4 cm; bodyweight: 89.0 ± 10.2 kg; and BMI: 27.3 ± 1.9. Prior to entering the saturation chamber, all participants underwent a medical examination by a trained health professional and body weight was also recorded to the nearest 0.1 kg (SECA, Birmingham, United Kingdom). The Norwegian Regional Committee approved the study protocol for Medical and Health Research Ethics (REK; approval number: 2018/1184). The participants provided their written informed consent before volunteering for the study.

### Saturation Dive Operation

Diving operations took place in October–November 2019, off the west coast of Norway and were conducted per the NORSOK U-100 requirements (Standards Norway, 2014). All participants were part of the same diving operation; therefore, they were exposed to similar dive conditions with a living depth of 72 metres seawater (msw) resulting in a maximum working depth of 81 msw. In the chamber the oxygen pressure was maintained at 380 mbar and this increased to 756 mbar during the bell run. The temperature was adjusted to maintain the thermal comfort of divers and typically maintained between 28 and 30°C. The dive operation was up to 18 days, with divers spending 14 days in a saturation at the living depth of 72 msw followed by a 3–4 days decompression period. The divers organised into 4 teams of 3 and working in overlapping 12 h shift patterns with a new 3-man shift starting every 6 h throughout a 24-h cycle. The divers had 12 h off between each shift. The dive teams worked in rotation; for every third shift, the divers provided support from the diving bell and did not perform any activities underwater. For the purposes of this study, an underwater excursion was defined the duration of a wet bell run during shifts where the divers undertook activity in the water, as one diver would remain dry in the bell throughout any given shift. Each diver completed 7.4 ± 1.7 underwater excursions on average during this operation, with an average underwater working period of 193.1 ± 25.9 min per excursion. The total time that a diver spent in water during this saturation operation was 1066.8 ± 417.9 min across the 21-day saturation dive. It was not possible to measure the underwater excursion’s intensity, but the divers subjectively reported that the underwater excursions were approximately half the length of the typical 5.30 h underwater working period, and the workload was perceived as “light.” Examples of work performed during underwater excursions during this operation include: Installation of blind flange plugs, pipe support installation, seal replacements, bell mouth installation, and inspection works.

### Measurement of Daily Energy Expenditure

The DLW stable isotope technique was used to measure energy expenditure. This is the gold-standard method that provides a safe and non-invasive procedure to determine energy expenditure (Westerterp, 2017). Participants completed a 10-day sampling period commencing 3 days after they were compressed to hyperbaric pressure equivalent to 72 msw. A second-morning void urine sample (∼ 1 ml) was collected to determine isotope analysis prior to the oral administration of a liquid bolus dose of hydrogen (deuterium ^2^H) and oxygen (^18^O) stable isotopes in the form of water (^2^H_2_^18^O). Additional water was added to the glass vials, and the participants were asked to drink this to ensure the full dose was ingested. A pre-prepared standardised dose, based on the weight of the subjects, was made in the Energetics Research Laboratory, Aberdeen and sent to the vessel to reduce any inconsistencies in dose preparation in the field. The dose was weighed to four decimal places. The prescribed dose was 10% ^18^O and 5% deuterium ^2^H based on a 90 kg participant using the following equation:

(1) $${}^{18}O\mathrm{dose}=\left[0.65\left(\mathrm{body}\mathrm{mass},g\right)\times \text{DIE}\right]/\text{IE}$$

where DIE is the desired initial enrichment (DIE = 618.923 × body mass (kg)−0.305) and IE is the initial enrichment (10%) 100,000 ppm.

**FIGURE 1:**
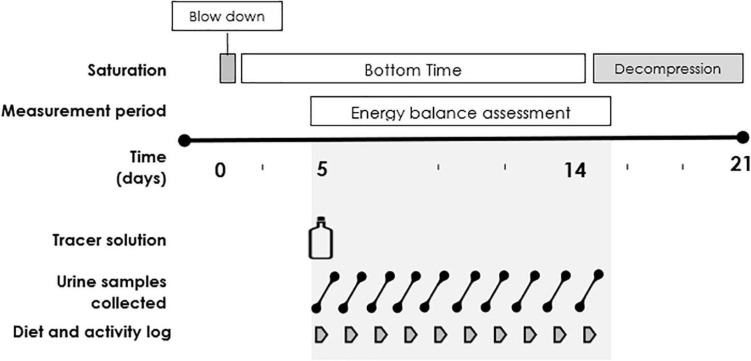
Outline of the study protocol.

Following the DLW administration, a morning urine sample (second void) was taken every 24 h for the subsequent 9 days (see Figure 1 for an overview of the study protocol) and kept in sealed containers. All urine samples were passed out of the saturation chamber *via* a hatch where they underwent decompression to the surface pressure. The urine containers were decompressed with unfastened lids, standing upright in a rack to avoid spillage. The lids were closed when the containers were collected from the hatch at surface pressure after decompression. The samples were subsequently frozen at −24 °C and stored on the vessel, before being transported to the laboratory in Aberdeen, United Kingdom, using a temperature-controlled cube (VeriCor Medical Systems, Holmen, WI, United States), where they remained frozen until analysis.

Analysis of the isotopic enrichment of urine was performed blind, using a Liquid Isotope Water Analyser (Los Gatos Research, United States) (Berman et al., 2012). Initially the urine was vacuum distilled, and the resulting distillate was used for analysis. Samples were run alongside five lab standards for each isotope and international standards to correct delta values to ppm. Daily isotope enrichments were log_*e*_ converted and the elimination constants (k_*o*_ and k_*d*_) were calculated by fitting a least squares regression model to the log_*e*_ converted data. The back extrapolated intercept was used to calculate the isotope dilution spaces (N_*o*_ and N_*d*_). A two-pool model, was used to calculate rates of CO_2_ production as recommended for use in humans (Speakman et al., 2021; Eq. 2).

(2) $$rCO_{2}=0.4554*N*\left[\left(1.007*K_{O}\right)-1.043*K_{d}\right)]*22.26$$

where k_*o*_ and k_*d*_ are in units of d^–1^ and rCO_2_ is in L d^–1^. Subsequently, rCO_2_ was used to determine DEE using the following equation:

(3) $$T\,E\,E=r\,C\,O_{2}*\left(1.106+\left(\frac{3.94}{0.85}\right)\right)*\left(\frac{4.194}{10^{3}}\right)$$

TEE is calculated in megajoules per day and subsequently converted into kcal per day for statistical analysis. For the purposes of this study an estimated respiratory quotient (RQ) of 0.85 was used, as this is often used during field-based studies where it is not possible to determine RQ (Butler et al., 2004). Furthermore, research suggests that despite a change in atmospheric conditions, expired O_2_ consumption and CO_2_ production (parameters used to calculate RQ) remain similar during a 17-day simulated saturation dive with a hyperoxic-helium gas mixture compared to surface ambient environment (Dressendorfer et al., 1977). An RQ of 0.85 was therefore deemed to be appropriate for analysis.

### Energy Intake

The kitchen on board the vessel controlled access to food. Participants were able to choose their diets from a daily menu throughout the day. The diver’s food choices and estimated portion sizes were recorded during the 10-day measurement period in a dietary log by the dive support team and researcher onboard. The dietary logs were assessed for completeness by a qualified nutritionist before analysis, with seven participants providing complete dietary logs that were subsequently analysed. A nutritional analysis software (Nutritics, United Kingdom) was used to determine the average daily energy intake during the 10-day measurement period.

### Statistical Analysis

Statistical analyses were performed using statistical software package SPSS (SPSS, version 22, IBM, United States). The Q–Q plots and Shapiro–Wilk test demonstrated that all variables were normally distributed. A paired *t*-test was used to test the difference between DEE and energy intake. Pearson’s correlation was conducted to assess the relationship between total time spent performing underwater activity during the measurement period and the DEE. Statistical significance was accepted at *p* < 0.05.

## Results

**FIGURE 2:**
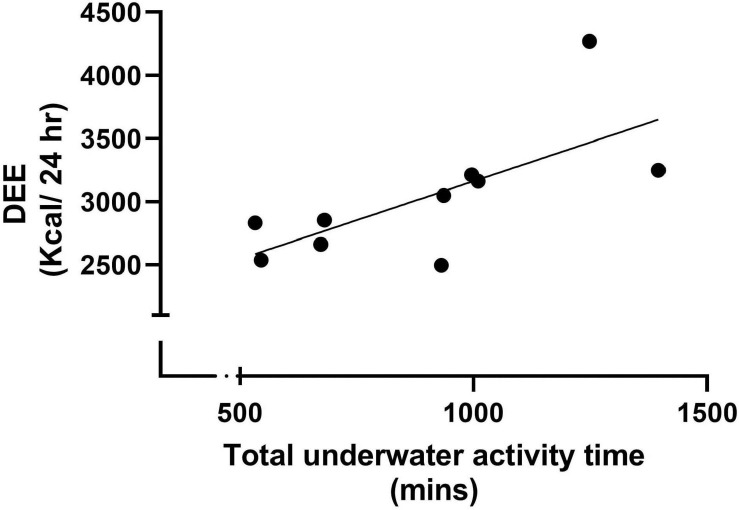
Shows the significant strong positive correction reported between DEE and total time spent performing underwater work (*r* = 0.7, *p* = 0.026).

During the 10-day measurement period participants complete on average 3.8 ± 1.2 underwater excursions, with a range from 2 to 6 excursions. Overall, participants spent 895.0 ± 273.8 min conducting wet bell runs, and each excursion was 237.8 ± 33.7 min on average. The mean actual DEE determined *via* DLW across the 10-day measurement period was 3030.9 ± 513.0 kcal/day, ranging from 2495 to 4268 kcal/day. When normalised for body mass, DEE was 32.5 ± 5.1 kcal/kg body mass/day and ranged between 28.6 and 40.1 kcal/kg body mass/day. Pearson’s correlation analysis revealed a significant strong positive correction reported between DEE and total time spent performing underwater work (*r* = 0.7, *p* = 0.026; Figure 2).

**FIGURE 3:**
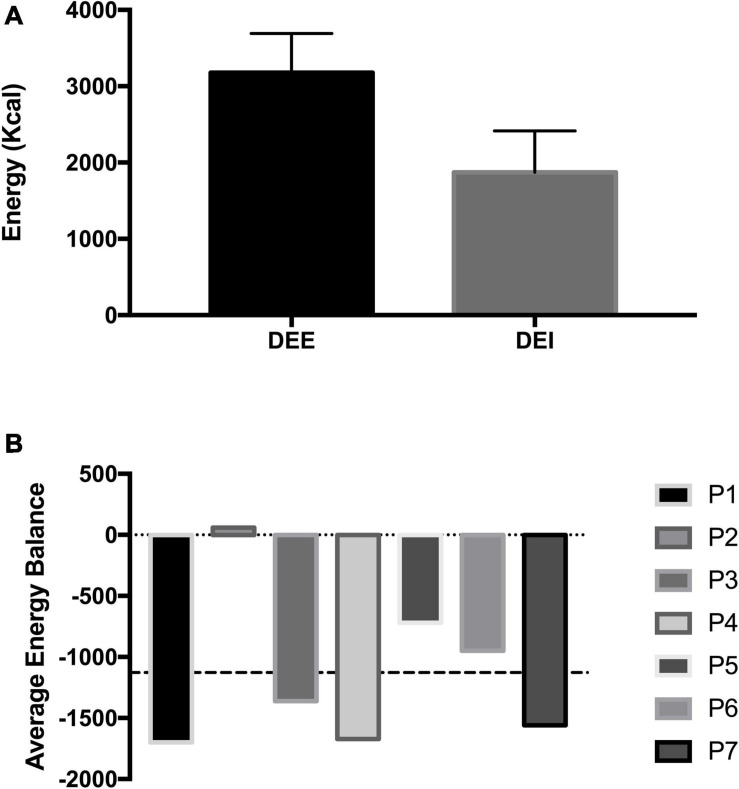
Panel **(A)** shows the mean daily energy expenditure (DEE) measured *via* DLW and mean daily energy intake (DEI) of saturation divers during a commercial operation in the North Sea. Panel **(B)** shows the individual energy balance (DEE–DEI) with commercial saturation divers. The dashed line represents the mean energy balance.

The average dietary intake was 1875.3 ± 487.4 kcal throughout the measurement period, which compared to actual energy expenditure, resulted in a significant 1021.1 ± 724.7 kcal negative energy balance (*p* = 0.005; Figure 3A). While this difference is substantial, one diver did consume enough calories during the measurement period, demonstrating that divers can meet the energy requirements of saturation diving (Figure 3B). Despite several divers not meeting the energy requirements during the 10-day measurement period, this was not reflected in body mass changes pre to post-dive. The diver’s average body mass remained unchanged.

## Discussion

This research study was the first to determine the average DEE of saturation divers during a live commercial diving operation in the North Sea. The data suggests a considerable individual variation in DEE between divers, with a range spanning 1745 kcal. This variability remained when DEE was normalised for body mass. There was a significant correlation between DEE and the total duration of underwater activity during the 10-day measurement period, with divers who spent more time in the water expending more energy. Energy intake was also reported to be significantly lower than average DEE, suggesting most of the divers did not attain an energy balance during the 10-day sampling period in saturation. The saturation divers and diving industry stakeholders should be aware that a divers’ dietary energy requirements must be personalised due to the intraindividual variability in DEE and energy intake in divers participating in the same diving operation.

While the current investigation reported a high DEE, it was not possible to establish if these requirements are more substantial than the demand within normobaric normoxic conditions (i.e., surface conditions) when physical activity and movement is matched. Early research by Seale et al. (1994) suggested that the hyperbaric heliox environment resulted in a significant DEE increase compared to the surface. Similarly, the authors utilised the DLW technique to determine DEE at the surface and hyperbaric conditions equivalent to 50 and 317 msw, using a saturation dive simulation without underwater excursions for 9 and 14 days, respectively. Participants had a significant 13 ± 4% increase in DEE at 50 msw compared to the surface, but no further significant DEE increases occurred with greater atmospheric pressure. The authors postulated that this increase might be explained by the hyperbaric atmosphere creating a greater gas density, thereby increasing airway resistance that results in more work required for respiration (Moon et al., 2009). Furthermore, helium (an inert gas required to create the hyperbaric environment) possesses a conductivity six times greater than air and subsequently increases heat exchange from the body (Hong et al., 2011). Together, these environmental factors may contribute to an increased DEE in commercial saturation divers.

This study observed that increases DEE were strongly correlated with total time spent conducting underwater activities, suggesting dive activities may be a primary determinant of DEE during commercial diving. The knowledge of physiological responses and intensities of underwater work for commercial saturation divers is limited. The physical and physiological demand of matched activity is however, greater in water compared to the surface due to the water density providing external resistance (Pendergast and Lundgren, 2009) and with greater depth the higher pressure increases the work of breathing (Moon et al., 2009). Within a commercial diving setting, saturation divers conduct several different tasks associated with the design and (de)construction of underwater structures, e.g., oil rigs. The intensity of the underwater work performed in this current study was reported to be low; however, previous reports of subjective workloads of different diving activities indicate certain activities require higher levels of endurance and strength (Gernhardt and Lambertsen, 2002). Therefore, the overall DEE in saturation divers is likely to be influenced by the intensity of underwater work, and a diver’s energy intake should be altered based on the task. There needs to be more research to understand the energy demand of various tasks that saturation divers perform underwater so that an accurate DEE can be calculated.

A theoretical analysis of calorie expenditure of open water diving in non-saturation divers suggested that dives that involve minimal movement may require 5.4 kcal/min with a range of between 3.8 and 8.1 kcal/min (Michniewski, 2020). With increased physical demand, this is suggested to go to 9.8 kcal/min and a range of 7.1–11.8 kcal/min. Consequently, providing an interesting insight into the energy demands of underwater activity; although, the differing equipment requirements and unique characteristics of saturation diving, these values cannot be directly applied. While underwater activity is likely to be a contributor to DEE, this study cannot discern the energy cost that is attributed to the resting metabolic rate (RMR) and activity-related expenditure. The RMR accounts for approximately 60% of DEE at sea level, contributing to a normal physiological process in a rested, post-absorptive and thermoneutral state (Pizzagalli et al., 2017). Given the change in atmospheric conditions, it is feasible that RMR during a saturation dive may also change compared to the surface. Further work should aim to measure the RMR of divers during hyperbaric hyperoxic exposure using indirect calorimetry. This will allow the prediction of energy expenditure during underwater activity to be calculated.

Knowledge of RMR may also help to explain some of the variability in DEE between divers. An individual’s weight and body composition influence their RMR, as a greater mass of metabolically active tissue (i.e. fat free-mass), results in a greater RMR (Amaro-Gahete et al., 2018). The participants’ body composition in the current study could not be measured, and further work should consider the influence of a diver’s body composition on the energy cost during a diving operation. In addition, those partaking in rotating shift patterns may also experience changes to their RMR and DEE as energy metabolism is subject to diurnal variation (Shaw et al., 2019). This study’s sample size was insufficient to discern any differences between divers on different shift patterns, but future research and nutritional guidelines for saturation divers should consider this.

During the saturation dive, dietary intake assessment revealed that divers consume significantly fewer calories than their DEE. While the current study is the first to demonstrate this during a commercial operation, existing research suggests that individuals may be prone to a negative energy balance during saturation diving simulations and have reported a reduced daily calorie intake (Webb et al., 1977; Collis, 1983; Smith et al., 2004). In the present study, this negative energy balance was not however, accompanied by a change in body weight, which is commonly cited to occur following commercial diving operations (Busch-Stockfish and Von Bohken, 1992). Theoretically, if a −1128 ± 725 kcal deficit was maintained throughout the 21-day saturation dive, a 3 kg weight loss may be expected. As weight did not change, it suggests that the calculated daily energy intake may be underestimated, which is a common error in dietary assessment research. In this study, however, dietary intake was restricted to the vessel’s food availability on a given day and food had to be passed into the chamber. As dietary logs were written as the divers were making their menu choices, this study did not rely on dietary recall or memory, which is cited as a source of error in dietary assessment (Naska et al., 2017). Equally, the energy expenditure and intake outside of the 10-day measurement period were not recorded; therefore, the energy balance across the full 21-day saturation dive is unknown, and the subsequent implications on body mass cannot be confirmed.

Maintaining an energy balance has important implications; however, saturation divers experience several unique food intake barriers. These include, but not limited to, the reduction in taste and palatability of certain foods, food availability based on differing shift patterns and a lack of appetite following underwater work (Deb et al., 2016). One participant in this study achieved an average energy balance, demonstrating that divers can attain their required energy intake. Nevertheless, further understanding is needed to identify the factors that influence a diver’s nutritional behaviours, so appropriate guidance and recommendations can be presented to the diving industry to account for the personalised needs of saturation divers.

The concept of personalised nutrition education uses information on individual characteristics to develop targeted nutrition advice, support or services that assist people in achieving a sustained behaviour change and improving health (Ordovas et al., 2018). In the context of saturation diving, these individual characteristics refer to unique challenges of their occupation and their onshore behaviours, preferences, and barriers to healthy eating. Furthermore, the on and off work schedule adds a further challenge to adopting a healthy lifestyle and nutrition habits. Research suggests that a sense of fatigue post saturation can last 1–10 days, with divers reporting varied timescales of returning to daily habits (Imbert et al., 2019). A personalised nutritional education approach should consider and encourage strategies that will help divers’ practice sustainable nutritional behaviours to help achieve energy balance during an operation and maintain their diet quality to promote cardiovascular health, body composition, and healthy ageing.

The current study presents a high degree of ecological validity, although the investigations’ field-based nature brings some limitations and opens additional research questions to address. Data in this study were collected in the same diving operation; therefore, all the divers were exposed to similar depths, conditions and work activities. Previous research in simulated conditions suggests that dive depth may not affect average DEE (Seale et al., 1994); this has not been tested in an operational setting within commercial saturation divers who perform underwater work. Equally, this study saw divers undertake an average of 7.4 underwater excursions across the whole dive, which is less than a typical operation. Given the activity underwater is a large part of a divers daily physical movement, other operations may result in a higher DEE.

Caution should be taken before applying the outcome of this study to the diving population, given the possibility of overestimating DEE due to confounding variables and the unique environmental conditions of saturation diving. For instance, the hyperbaric hyperoxic-helium environment may increase RQ above the estimated 0.85 used to determine DEE. Little is known about the metabolic changes during saturation diving; however, evidence suggests that changes in lipid metabolism are non-significant during experimental saturation dives at 70 m. Although during deeper dives to <200 m, changes in lipid metabolism are expressed through changes in blood metabolites (Buravkova and Popova, 2007). Divers in the current study had a living depth of 72 msw and a maximum working depth of 81 msw, suggesting changes in lipid metabolism, and therefore an increased RQ, is less likely. Future studies should consider indirect calorimetry during saturation exposure to allow direct assessment of RQ and therefore, increase the accuracy of DEE calculations *via* DLW. Equally, future studies may consider monitoring body composition pre and post dive using accurate techniques, such as dual X-ray absorptiometry (DEXA), which can help identify if the negative energy balance resulted in changes in body fat percentage and muscle mass of the divers. No changes in body weight were observed in the current study and while this may be explained by increased fluid intake to account for any loss in body weight, this study did not monitor fluid intake. As such, this study cannot conclude with certainty that saturation divers experience a negative energy balance during a commercial operation due to the potential overestimation in DEE or underestimation of DEI. Nevertheless the findings in this study corroborate previous research during simulated saturation dives that indicate divers may experience an elevated DEE (Seale et al., 1994) and may also reduce DEI (Webb et al., 1977; Collis, 1983; Smith et al., 2004).

## Conclusion

In conclusion, this study is the first to assess the energy expenditure of saturation divers during a commercial saturation dive using DLW. Considerable variability in DEE was reported, along with a mismatch between DEE and energy intake during the 10-day measurement period. This suggests that the dietary intake requirements and practices can vary between divers, and a personalised approach to nutritional intake may be needed. The study outcomes are drawn from a single diving operation, where all participants performed similar dive activities and exposed to similar environmental conditions. Further offshore field research should aim to understand the causes of DEE variability and the factors that may influence a diver’s nutritional intake. Saturation divers have a unique nutritional requirement due to occupational demands. As such, the dietary recommendations for saturation divers must be cognisant of these factors to promote their health and well-being. Addressing these factors requires continued personalised nutrition support from the diving industry and contractors to ensure meaningful and sustainable changes.

## Data Availability Statement

The raw data supporting the conclusions of this article will be made available by the authors, without undue reservation.

## Ethics Statement

The studies involving human participants were reviewed and approved by the Norwegian Regional Committee approved the study protocol for Medical and Health Research Ethics (REK; approval number: 2018/1184). The patients/participants provided their written informed consent to participate in this study.

## Author Contributions

SD and IE contributed to the conception and design of the study. IE performed the data collection. CH and JS conducted the DLW analysis. SD wrote the first draft of the manuscript. All authors contributed to manuscript revision, read, and approved the submitted version.

## Conflict of Interest

SD was awarded funding by Equinor which funds part of this research study. IE was funded Norwegian Research Council and Equinor on behalf of PRSI Pool (Petromaks2 project no. 280425). OE is currently employed by Equinor. The conception, design and analysis of the study were conducted independently of the funder with no commercial involvement. The remaining authors declare that the research was conducted in the absence of any commercial or financial relationships that could be construed as a potential conflict of interest.
